# GABA accretion reduces Lsi-1 and Lsi-2 gene expressions and modulates physiological responses in *Oryza sativa* to provide tolerance towards arsenic

**DOI:** 10.1038/s41598-017-09428-2

**Published:** 2017-08-18

**Authors:** Navin Kumar, Arvind Kumar Dubey, Atul Kumar Upadhyay, Ambedkar Gautam, Ruma Ranjan, Saripella Srikishna, Nayan Sahu, Soumit Kumar Behera, Shekhar Mallick

**Affiliations:** 1 CSIR-National Botanical Research Institute, Lucknow, India; 2 Department of Biochemistry, Faculty of Science, Banaras Hindu University, Varanasi, India

## Abstract

GABA counteracts wide range of stresses through regulation of GABA shunt pathway in plants. Although, GABA assisted tolerance against As toxicity in plants is still unexplored. We have examined GABA induced tolerance in rice seedlings with two exposure periods of GABA i.e., short term and long term. Results showed that accumulation of GABA reduced the expressions of Lsi-1 and Lsi-2 transporter genes, which ultimately decreased the accumulation of As in rice seedlings. The accumulation of GABA also modulated the gene expression of GABA shunt pathway and activity of antioxidant enzymes, which strongly induced the tolerance in plants. Antioxidant enzymes such as CAT, POD, GPX and SOD showed maximum alteration in activity with GABA accretion. In both exposure periods, long term accumulation of GABA was highly efficient to provide tolerance to plants against As(III), while higher level of GABA at short term was toxic. Tolerance responses of GABA towards As(III) was reflected by minimal changes in various physiological (WUE, *A*, gs, PhiPS2, qp, NPQ, ETR and Trmmol) and growth parameters with concomitant accumulation. Oxidative stress marker such as TBARS and H_2_O_2_ contents were reduced with GABA accumulation. These results suggested that GABA sturdily inhibits As accumulation and provides tolerance towards As(III).

## Introduction

γ-Aminobutyric acid (GABA) is a non-protein four carbon amino acid, which counteract instantly against biotic and abiotic stresses in plants^1, 2^. It is metabolized via GABA shunt pathway in plants, including three enzymes i.e., glutamate decarboxylase (GAD), GABA transaminase (GABA-T) and succinic semialdehyde dehydrogenase (SSADH)^3–5^. The GABA shunt is also known to assimilate carbon from glutamate and in generation of C:N fluxes which employ in tricarboxylic acid (TCA) cycle. The two enzymes *viz*., succinyl CoA ligase and α-ketoglutarate dehydrogenase of TCA cycle are very sensitive to reactive oxygen species (ROS) and inhibited during oxidative stress^1^. The inhibited intermediate of TCA cycle i.e., succinate, replenished by the GABA shunt pathway^6, 7^. GABA also modulates the accumulation of Ca^2+^ in the cell, which, enhances the activity of GAD and accumulation of endogenous GABA^8^. In a recent study, GABA is reported to enhance antioxidant levels in aluminum treated barley seedlings^9^. Similarly, GABA supplementation has also been demonstrated against Ca(NO_3_)_2_ stress through regulating polyamines and GABA metabolism in muskmelon seedlings^10^. However, GABA signaling modulates plant growth by directly regulating the activity of plant-specific anion transporters and cytosolic pH^2^. GABA, not only triggers cell signaling, but also regulates expression of stress and growth responsive genes of plants^11, 12^. In abiotic stress conditions, GABA plays crucial role to ameliorate environmental stress through increasing its concentration against drought, heat, salt and transient environmental factors, including touch, wind, rain, etc.^7, 13, 14^. In addition, GABA enhances the antioxidants activities in the plants to maintain the threshold level of ROS in the cell against abiotic stress^9^.

GABA as a signal molecule, up-regulates nitrate (NO_3_^−^) uptake, metabolism and nitrogen transport in plants^15, 16^. Also, the signaling molecule, salicylic acid (SA) is reported to ameliorate As toxicity in rice seedlings by reducing its translocation in root to shoot involving nitric oxide (NO) as a signalling molecule^17^. Similarly, NO has also been reported to protect plants from As induced oxidative damage^18^. Both the molecules i.e., SA and NO are reported to be interlinked which eventually triggers the NPR1 (Nonexpresser of Pathogenesis-Related genes1) mediated pathway for systemic acquired resistance in plants under stress^19^. Hence, in light of the reports of the GABA mediated amelioration of abiotic stress in plants, it is speculated that it can simultaneously regulate different pathways of cell metabolism and stress responsive activities, which are largely unclear.

Other stresses, including cold, heat, salt, and mild or transient environmental factors, such as touch, wind, rain, etc. rapidly increase cellular levels of Ca^2+^. Increased cytosolic Ca^2+^ stimulates calmodulin-dependent glutamate decarboxylase activity and GABA synthesis. A review of the kinetics of GABA accumulation in plants reveals a stress-specific pattern of accumulation that is consistent with a physiological role for GABA in stress mitigation. Recent physiological and genetic evidence indicates that plants may possess GABA-like receptors that have features in common with the animal receptors. The mechanism of action of animal GABA receptors suggests a model for rapid amplification of ion-mediated signals and GABA accumulation in response to stress. Metabolic pathways that link GABA to stress-related metabolism and plant hormones are identified. The survival value of stress-related metabolism is dependent on metabolic changes occurring before stress causes irreversible damage to plant tissue. Rapid accumulation of GABA in stressed tissue may provide a critical link in the chain of events leading from perception of environmental stresses to timely physiological responses.

On the other hand, heavy metal such as arsenic (As), cadmium (Cd), mercury (Hg) and lead (Pb), causes food contamination through its accumulation in edible parts of the crops. However, arsenic (As) is the serious global concern, especially dire in the South East Asian countries^20, 21^. These regions cultivate 90% rice of the world. The way of rice cultivation, harbors As in the grain, causes food chain contamination, resulting into acute As health risks^22^. Watanabe *et al*.^23^ reported that the accumulation of As, can alter the uptake of the minerals and reduce the crop yields. Plants generally accumulate two forms of As [arsenate (AsV) and arsenite As(III)], in which As(III) is more toxic^24^. Arsenate is a phosphate analogue and transport in plant through specific phosphate transporters^25^. However, entry of As(III) is facilitated via Lsi-1 and Lsi-2 transporters located on the root of plants^26^. Although, there is no known role of As in plants, but As(III) very efficiently accumulates in plants from soil, causes oxidative damage through ROS burst^27^. Reactive oxygen species are highly reactive molecules, which disrupt physiological responses, biochemical activities and metabolism of plants^28^. Several signaling molecules have been reported to ameliorate As toxicity in plants. Although, the role of GABA in amelioration of As induced stress in plants remains largely unexplored.

In the light of these findings, we hypothesized this study with following questions. Does GABA provide tolerance against As(III) stress? Whether, GABA regulates the gene expressions of As transporters and accumulation towards As(III) toxicity in plants.

## Results

### GABA application reduces As accumulation and recovers growth from As(III) stress

The application of GABA enhances endogenous level of GABA and modulates metal accumulation in plants^2, 9^. In present study, the treatment of GABA (50 and 100 µg ml^−1^) elevated the endogenous level of GABA and simultaneously reduced the As accumulation in rice seedlings (Table 1). However, the rice seedlings treated with As(III) only elevated the level of GABA in root (45%) and shoot (55%), respectively, over the control. In both the exposure periods (long term and short term), long term GABA was more effective in reduction of As accumulation. The maximum reduction in As accumulation i.e., 54% in shoot and 67% in root were observed in As(III) + GABA(L) (Long term) and As(III) + GABA(H) (Long term), respectively, in comparison to As(III).

**Table 1:** Accumulation (µg g^−1^ dw) of As and GABA (nM g^−1^ fw) in the shoot and root of *Oryza sativa* L. cv.

Accumulation				
Treatments	As		GABA	
	Shoot	Root	Shoot	Root
C	—	—	241.84 ± 11.82^a^	116.03 ± 10.36^a^
GABA(L) (Long term)	—	—	275.35 ± 8.49^b^	269.24 ± 13.98^c^
GABA(H) (Long term)	—	—	405.98 ± 8.04^f^	479.80 ± 41.72^e^
GABA(L) (Short term)	—	—	259.18 ± 9.97^ab^	191.94 ± 14.41^b^
GABA(H) (Short term)	—	—	308.31 ± 2.85^c^	250.54 ± 8.15^c^
As(III)	19.04 ± 1.02^d^	166.31 ± 4.32^d^	375.18 ± 6.48^e^	167.88 ± 15.16^ab^
As(III) + GABA(L) (Long term)	8.65 ± 0.56^a^	54.16 ± 3.75^a^	324.60 ± 8.49^cd^	144.68 ± 17.08^ab^
As(III) + GABA(H) (Long term)	10.27 ± 0.45^ab^	53.82 ± 5.59^a^	332.34 ± 2.83^d^	173.40 ± 18.47^ab^
As(III) + GABA(L) (Short term)	14.36 ± 0.33^c^	84.93 ± 2.72^b^	268.66 ± 3.28^b^	260.62 ± 8.09^c^
As(III) + GABA(H) (Short term)	16.59 ± 1.49^cd^	100.55 ± 4.43^bc^	437.54 ± 10.65^g^	392.27 ± 53.37^d^

Pant-10 after long term and short term GABA treatments. Values marked with same alphabets are not significantly different (DMRT, p < 0.05). All the values are means of four replicates ± SD.

The growth parameters were assessed to study the role of GABA against As(III) stress in rice seedlings. The growth of rice seedlings were enhanced with accumulation of GABA in both exposure periods (Table 2). GABA(L) at long term significantly (~11%) enhanced the growth parameters (shoot length and fresh weight), as compared to control. However, As(III) treated seedlings exhibited reduction in growth parameters (shoot length 14% and fresh weight 8.5%). The application of GABA for long term in As(III) recovered the growth, whereas, short term was not effective. Although, the root length of rice seedlings treated with As(III) was increased, as compared to the control, which was normalized towards control by the application of GABA. The study reveals that the accumulation of GABA antagonizes the As accumulation and recovers the growth.

**Table 2:** Effect of GABA and As(III) accumulation in shoot, root lengths (cm), fresh-weight (g), ascorbic acid (µM g^−1^ FW), TBARS content (nM g^−1^ FW), H_2_O_2_ (nM g^−1^FW) and GSH/GSSG ratio of *Oryza sativa* cv. Pant-10 leaves.

Treatments	Shoot lengths	Root lengths	Fresh-weight	Ascorbic acid	TBARS	H_2_O_2_	GSH/GSSG
C	26.75 ± 0.50^bc^	6.75 ± 0.29^a^	11.93 ± 0.74^bcd^	30.29 ± 3.06^a^	105.33 ± 9.66^ab^	464.90 ± 13.17 ^cd^	1.27 ± 0.08^a^
GABA(L) (Long term)	29.67 ± 0.58^d^	9.63 ± 0.75^ef^	13.21 ± 0.84^cd^	79.37 ± 8.23^b^	98.38 ± 4.43^a^	393.17 ± 45.37^abc^	2.50 ± 0.17 ^cd^
GABA(H) (Long term)	29.00 ± 2.00^cd^	8.75 ± 0.50^cde^	11.63 ± 0.72^bc^	24.25 ± 2.43^a^	97.34 ± 8.56^a^	339.64 ± 56.79^ab^	1.70 ± 0.20^b^
GABA(L) (Short term)	26.75 ± 1.50^bc^	10.13 ± 0.85^f^	11.65 ± 0.84^bc^	149.79 ± 24.03^c^	105.81 ± 6.57^ab^	337.72 ± 17.24^ab^	2.68 ± 0.15^de^
GABA(H) (Short term)	27.50 ± 2.89^bcd^	9.38 ± 0.48^ef^	13.29 ± 1.00^cd^	69.11 ± 8.18^b^	105.31 ± 9.29^ab^	469.21 ± 78.46 ^cd^	1.73 ± 0.14^b^
As(III)	22.88 ± 0.35^a^	9.25 ± 0.87^def^	10.93 ± 0.24^ab^	143.75 ± 18.45^c^	141.64 ± 7.60^d^	528.66 ± 17.34^de^	2.99 ± 0.32^e^
As(III) + GABA(L) (Long term)	26.25 ± 2.04^b^	9.00 ± 0.41^cde^	12.66 ± 0.94^bcd^	212.37 ± 5.05^d^	118.58 ± 1.28^bc^	427.99 ± 40.18^bc^	2.16 ± 0.26^c^
As(III) + GABA(H) (Long term)	25.50 ± 1.29^b^	8.25 ± 0.50^bc^	13.63 ± 1.67^d^	59.85 ± 3.12^ab^	123.18 ± 14.08^c^	306.20 ± 17.92^a^	2.16 ± 0.27^c^
As(III) + GABA(L) (Short term)	25.33 ± 1.15^b^	8.33 ± 0.58^bcd^	12.14 ± 0.52^bcd^	90.08 ± 8.42^b^	97.80 ± 12.31^a^	418.02 ± 65.68^bc^	2.66 ± 0.04^de^
As(III) + GABA(H) (Short term)	21.75 ± 0.96^a^	7.50 ± 0.41^ab^	9.89 ± 1.05^a^	133.77 ± 7.11^c^	127.48 ± 0.73^cd^	564.76 ± 46.49^e^	2.27 ± 0.21^c^

Values marked with same alphabets are not significantly different (DMRT, p < 0.05). All the values are means of four replicates ± SD.

### GABA reduced the expression of Lsi-1 and Lsi-2 transporters

**Figure 1:**
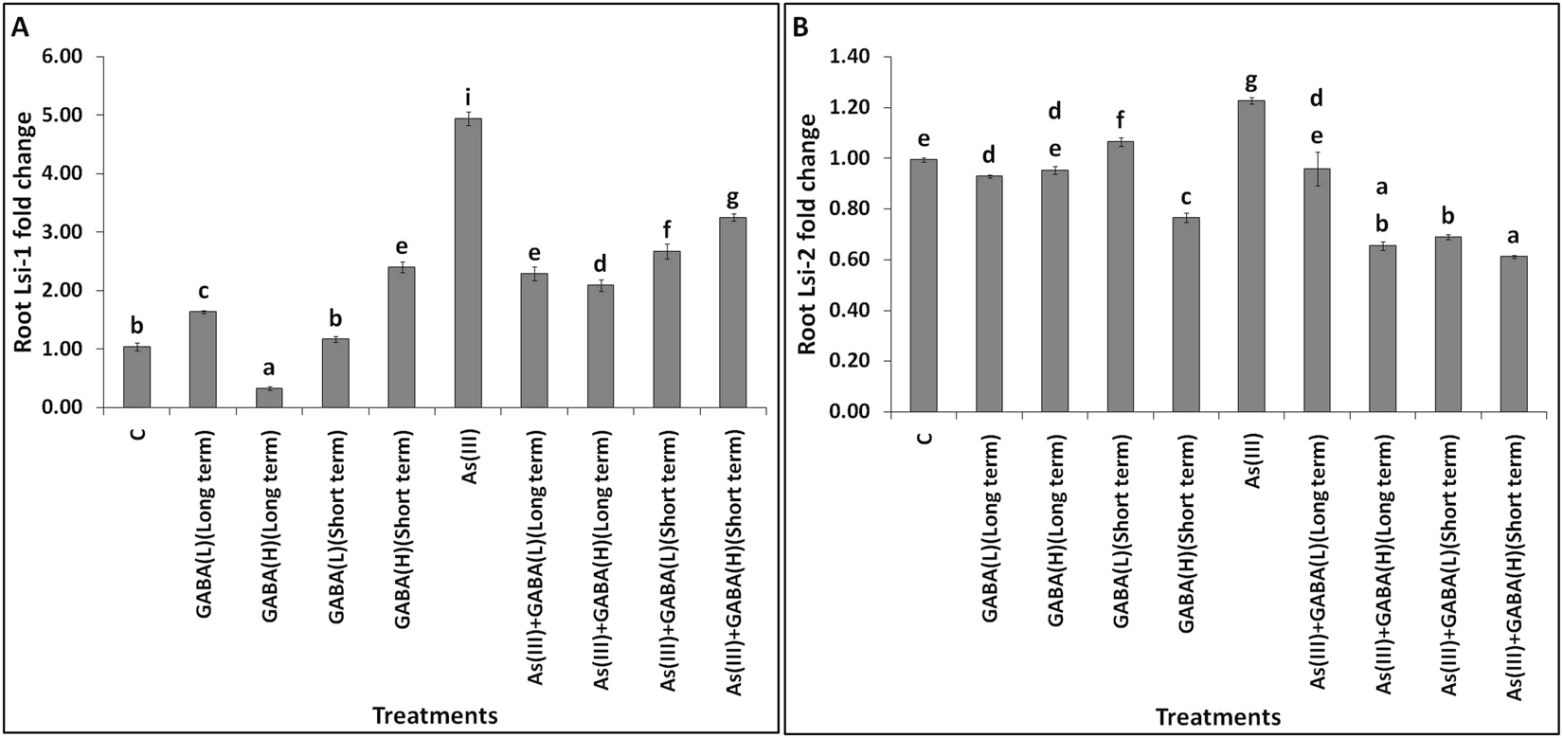
Gene expression of As(III) specific transporters [Lsi-1 (**A**) and Lsi-2 (**B**)] in root of rice seedlings treated with GABA and As(III). All the values are presented as mean ± SD (n = 4).

GABA signaling can regulate several gene expressions in plants to improve growth^2^. In the present study, the application of GABA alone induced the expression of Lsi-1 in the roots, except for higher dose of GABA at long term exposure period (Fig. 1A). Further, rice treated with As(III) significantly (~5 fold) enhanced the expression of Lsi-1 in root as comparison to control. However, the application of GABA significantly reduced the expression of Lsi-1, in which long term treatment was more effective than the short term. The expression of Lsi-2 showed non-significant change with GABA application, except it was reduced with GABA(H) at short term (Fig. 1B). The treatment of As(III) significantly enhanced the expression of Lsi-2 (1.2 fold) in root as compared to the control. However, the application of GABA significantly reduced the expression of Lsi-2, where GABA(H) was more effective than GABA(L) in the reduction of its expression.

### GABA treatment recovers the physiological parameters from As(III) stress

**Figure 2:**
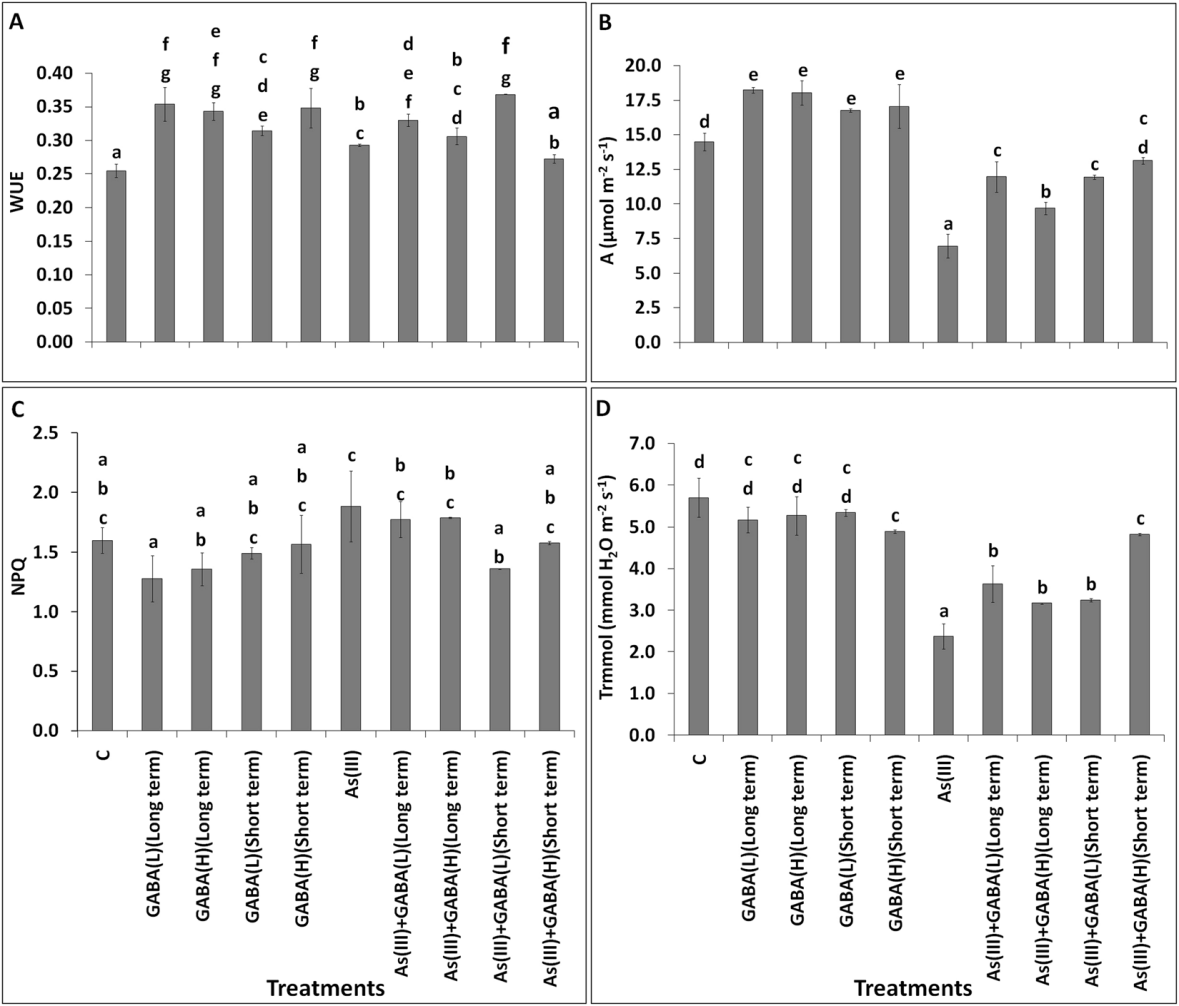
Effect of GABA accumulation on (**A**) Water use efficiency (WUE), (**B**) Photosynthetic carbon assimilation rate; (**A**), (**C**) Non-photochemical quenching (NPQ) and (**D**) Leaf transpiration (Trmmol) in the leaves of the *Oryza sativa* L. with As(III) treatments. Values marked with same alphabets are not significantly different (DMRT, p < 0.05). All the values are means of four replicates ± SD.

The application of GABA reduces the toxicity symptoms of the plants, suffering from biotic and abiotic stress^1^. In our study, the physiological parameter such as water use efficiency (WUE) of rice plants was enhanced with all the GABA treatments either with or without As(III) (Fig. 2A). However, WUE of plants under As(III) treatment was marginally decreased as compared to GABA treatments. However, photosynthetic carbon assimilation rate (A) was enhanced in seedlings applied with GABA, with or without As(III) as compared to their respective control (Fig. 2B). The maximum assimilation rate was observed with As + GABA(H) applied for short term, over the As(III). Similar to the A, same trends were also observed with the other physiological parameters *viz*., stomatal conductance (gs), fraction of absorbed photons (PhiPS2), photochemical quenching (qP) and electron transport rate (ETR), over the control and As(III) (Supplementary Fig. 2C–F), except for gs under GABA treatment. However, non-photochemical quenching (NPQ) and leaf transpiration (Trmmol) were reduced with GABA treatments, as compared to control (Fig. 2G). Further, reduction in Trmmol 58% was also observed with As(III) treatment (Fig. 2H). However, Trmmol was enhanced by the application of GABA and As(III). The maximum enhancement in Trmmol level i.e., 103% was observed in As(III) + GABA(H) for short term, compared to the As(III). On the contrary, NPQ was observed to be reduced with GABA treatments and enhanced with As(III). The higher reduction in NPQ was observed with As(III) + GABA(L) (Short term), as compared to As(III). The study, suggested that application of GABA enhances the growth and recovers physiological parameters of plants during As stress.

### GABA accumulation simultaneously reduces lipid peroxidation and increases antioxidant activities

The level of TBARS and H_2_O_2_ are marker of cellular toxicity which increases during oxidative stress^29^. In the present study, the treatment of As(III), increased the TBARS content (34%) in rice plants, in comparison to the control. However, reduction in TBARS content was observed in all the treatments of GABA and As(III) combinations (Table 2). Significant reduction in TBARS content was observed with the GABA(L) either with short term or long term treatments, as compared to As(III). Similarly, the level of H_2_O_2_ was decreased in all the GABA alone treatments and with As(III) combinations, except for GABA(H) at short term (Table 2).

The antioxidant system of the plants maintains the level of ROS in the cell^29^. The antioxidant, ascorbate content was increased in all the treatments as compared to control (Table 2). The GABA(L) at short term was more effective in increasing of ascorbate content. Further, all the treatments of GABA applied with As(III) also increased the plant ascorbate content. In the case of As(III) + GABA(L) treatment, the ascobate content was highly increased in the seedlings, as compared to all the treatments of GABA and As(III). The antioxidant enzymes involved in detoxification of As(III) induced oxidative stress, exhibited higher activities with GABA accumulation (Table 3). The activities of APX, GPX, DHAR and MDHAR were positively increased in GABA(L) treatment at short term (Table 3). However, the activities of POD and SOD in GABA(H) at short term, were higher under As(III) stress except CAT. Similarly, the activities of DHAR and MDHAR were higher in As(III) + GABA applied for long term. However, the activity of GR recovered upto the level of control with treatment of GABA(L). On the contrary, activity of GST did not increase significantly in all the treatments except in case of GABA(L) at short term. The ratio of GSH/GSSG was higher in all the treatments as compared to control (Table 2). GSH/GSSG ratio in GABA(L) treated plants was higher than GABA(H) treatments. The treatment of As(III), significantly increased the ratio of GSH/GSSG. However, the application of GABA with As(III) reduced the ratio of GSH/GSSG in seedlings, as compared to the As(III). Overall, results demonstrate that the application of GABA reduces the oxidative stress marker and modulates the antioxidant defense system towards stress.

**Table 3:** Effect of GABA accumulation on different antioxidant enzymes [CAT: Catalase (mM mg^−1^ P); APX: Ascorbate peroxidase (µM mg^−1^ P); GPX: Glutathione peroxidase (µM mg^−1^ P), SOD: Superoxide dismutase (U mg^−1^ P); POD: Guaiacol peroxidase (mM mg^−1^ P); DHAR: Dehydroascorbate reductase (µM mg^−1^ P); MDHAR: Mono-dehydroascorbate reductase (µM mg^−1^ P); AO: Ascorbate oxidase (U mg^−1^ P); GR: Glutathione reductase (U mg^−1^ P); GST: Glutathione S-transferase (µM mg^−1^ P)] activities in As(III) treated and untreated seedlings of *Oryza sativa* cv.

Treatments	CAT	APX	GPX	SOD	POD	DHAR	MDHAR	AO	GR	GST
C	4.87 ± 0.66 ^cd^	39.82 ± 5.84^bcd^	10.34 ± 0.14^bc^	5.02 ± 0.14^ab^	0.54 ± 0.04^cde^	22.93 ± 2.77^a^	9.31 ± 0.36 ^cd^	12.41 ± 1.65^ab^	43.70 ± 2.68^abc^	3.92 ± 0.02^ab^
GABA(L) (Long term)	5.87 ± 0.39^e^	44.71 ± 7.97^cde^	11.88 ± 0.79 ^cd^	4.51 ± 0.38^a^	0.50 ± 0.06^bc^	28.59 ± 3.27^ab^	9.09 ± 0.37^c^	16.85 ± 0.47^cdef^	50.48 ± 2.92 ^cd^	3.82 ± 0.14^a^
GABA(H) (Long term)	5.15 ± 0.55^de^	40.31 ± 2.54^bcd^	10.88 ± 1.05^bcd^	5.13 ± 0.53^bc^	0.61 ± 0.06^e^	30.94 ± 0.64^b^	10.06 ± 0.38 ^cd^	19.42 ± 1.75 ^f^	50.73 ± 5.04 ^cd^	4.46 ± 0.62^abcd^
GABA(L) (Short term)	4.82 ± 0.63^bcd^	51.96 ± 6.18^e^	12.13 ± 0.78^d^	5.39 ± 0.28^bcd^	0.52 ± 0.02^bcd^	38.95 ± 3.91 ^cd^	12.10 ± 0.33^e^	19.20 ± 0.50^ef^	50.28 ± 6.55 ^cd^	5.29 ± 0.24^d^
GABA(H)(Short term)	4.37 ± 0.14^abcd^	36.41 ± 1.24^abc^	10.40 ± 0.12^bc^	5.75 ± 0.44^de^	0.54 ± 0.05^cde^	33.09 ± 0.44^bc^	10.58 ± 1.37^d^	17.67 ± 1.06^def^	44.17 ± 4.67^abc^	4.03 ± 0.36^abc^
As(III)	4.19 ± 0.23^abc^	44.97 ± 3.29^cde^	11.94 ± 0.47 ^cd^	5.87 ± 0.13^de^	0.60 ± 0.04^de^	40.05 ± 4.99 ^cd^	6.35 ± 0.91^b^	11.32 ± 1.25^a^	56.43 ± 5.04^d^	4.89 ± 0.40^bcd^
As(III) + GABA(L) (Long term)	4.01 ± 0.32^abc^	30.26 ± 1.54^ab^	14.10 ± 0.39^e^	5.71 ± 0.25^cde^	0.45 ± 0.06^ab^	52.05 ± 5.30^e^	7.43 ± 0.05^b^	16.36 ± 2.40^cde^	36.63 ± 1.26^a^	4.44 ± 0.59^abcd^
As(III) + GABA(H) (Long term)	3.44 ± 0.45^a^	47.50 ± 1.37^de^	11.67 ± 1.17 ^cd^	5.30 ± 0.16^bcd^	0.47 ± 0.04^abc^	43.72 ± 0.29^d^	13.64 ± 0.53 ^f^	14.97 ± 1.54^bcd^	45.55 ± 4.88^bc^	4.99 ± 0.47 ^cd^
As(III) + GABA(L) (Short term)	4.30 ± 0.48^abcd^	27.73 ± 1.57^a^	9.28 ± 0.17^b^	5.56 ± 0.40^bcd^	0.40 ± 0.05^a^	34.65 ± 3.63^bc^	6.31 ± 0.77^b^	13.12 ± 0.05^ab^	37.76 ± 3.60^ab^	4.03 ± 0.38^abc^
As(III) + GABA(H) (Short term)	3.91 ± 0.51^ab^	33.13 ± 0.33^ab^	7.30 ± 0.74^a^	6.19 ± 0.52^e^	0.60 ± 0.01^de^	44.79 ± 5.94^d^	4.88 ± 0.29^a^	14.48 ± 2.13^bc^	56.20 ± 3.47^d^	4.33 ± 0.54^abcd^

Pant-10. Values marked with same alphabets are not significantly different (DMRT, p < 0.05). All the values are means of four replicates ± SD.

### GABA accumulation modulated the GABA shunt gene expression

The application of exogenous GABA modulates the expression of genes, involved in the cellular metabolism^5^. In present study, the application of GABA(L) at long term significantly enhanced the expressions of succinyl CoA ligase (S. CoA ligase) and α-ketoglutarate dehydrogenase (α-KDH) in shoot, over the control (Supplementary Fig. 1A–D). However, the expression of S. CoA ligase gene was reduced and α-KDH gene enhanced with GABA(H) treatment at short term. Similarly, the expressions of shoot S. CoA ligase in As(III) treated rice seedlings were decreased by 0.80 fold, as compared to control. On the contrary, the expression of shoot S. CoA ligase and α-KDH in seedlings treated with As(III) + GABA(L) at long term were enhanced while reduced with GABA(H). In root, the expression of S. CoA ligase enhanced significantly with GABA(L) while reduced with GABA(H) as respect to the control. However, the expression of α-KDH reduced in all the treatments of GABA controls. In case of As(III) treatment, slight reduction in the expression of S. CoA ligase and α-KDH (0.634 fold) were observed, in the root, as respect to control, whereas, recovery in expression was observed with application of GABA. The expression of succinate dehydrogenase (SDH) in root and shoot was enhanced with the GABA(L) treatment at short term in comparison to the control (Supplementary Fig. 1E,F). Similarly, expression of SDH in As(III) treated seedlings was also slightly enhanced in root and shoot. Further, enhancement in the expression of SDH was observed with the GABA(L) treatment applied for long term.

**Figure 3:**
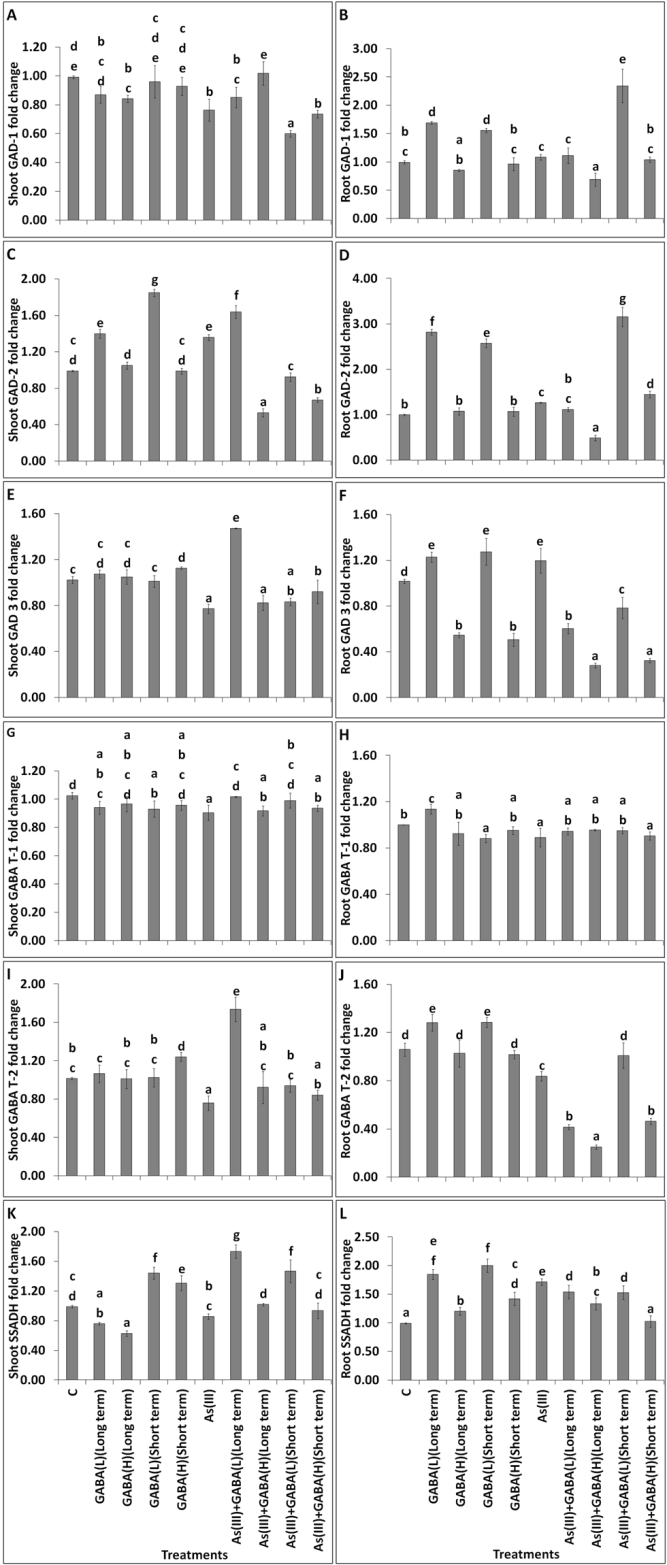
Effects of GABA accumulation on gene expressions involved in GABA shunt pathway [GAD: Glutamate decarboxylase (**A**–**F**), GABA T: GABA transaminase (**G**–**J**), SSADH: Succinyl semialdehyde dehydrogenase (**K**,**L**)] with As(III) treated *Oryza sativa* L. All the values are means of four replicates ± SD.

The first gene of GABA shunt pathway i.e., GAD exists in several isoforms, in which the expression of shoot (Fig. 3A,E) GAD1 decreased with GABA(H) at long term and GAD 3 reduced with GABA(H) short term as compared to control. However, the expression of GAD 2 was highly enhanced with the GABA(L) in shoot at both the exposure periods. The treatment of As(III), slightly reduced the expressions of GAD 1 and GAD 3 in shoots in comparison to the control, while enhanced in the case of GAD 2. The treatment of GABA(L) with As(III) enhanced the expression of shoot GAD1, GAD 2 and GAD 3 in comparison to rice treated with As(III) (Fig. 3A,C,E). However, in rest of the GABA applications, the expression of GAD isoforms were reduced in the shoot, except for GAD1 expression enhanced with As(III) + GABA(H) (Long term) treatment. In root, the expressions of GAD genes were significantly enhanced with the GABA(L) at both the exposure periods. Similarly, the expressions of GAD 1, GAD 2 and GAD 3 were induced with As(III) treatment (Fig. 3B,D,F). The expressions of GAD 1 and GAD 2 were significantly enhanced with the As(III) + GABA (L) for short term, except GAD 3. The second gene of GABA shunt, GABA T exists in two isoforms, in which the expressions of shoot GABA T1 and T2 showed non-significant change with the application of GABA, except for GABA T2, which was enhanced with application of GABA(H) for short term (Fig. 3G,I). In case of As(III) treated rice seedlings, the expression of shoot GABA T1 and T2 were slightly reduced, as compared to the control. However, the expression of all GABA T1 and T2 in the rice shoots treated with GABA(L) at long term were enhanced, in comparison to the As(III). GABA T1 expression in the roots showed no significant change with As(III) + GABA treatment, except with GABA(L) for long term where it increased, in comparison to control. However, the expression of root GABA T2 was enhanced significantly with GABA(L) at both the exposure periods. A significant reduction in expression of root GABA T2 was observed in seedlings treated with As(III). Further, reduction in the expression was also observed in As(III) + GABA, except in As(III) + GABA(L) for short term. The third gene of GABA shunt pathway, succinic semialdehyde dehydrogenase (SSADH) expression in shoot was reduced significantly with the long term GABA application, while it was enhanced with the short term in comparison to the control (Fig. 3K). A slight reduction in the expression of shoot SSADH was observed with the As(III). However, the combination of As(III) + GABA(L) significantly induced the expression of SSADH in shoot, as respect to the As(III). In root, SSADH expression was significantly enhanced with all the GABA application at both the exposure periods (Fig. 3L) as compared to the control. In the case of rice seedling treated with As(III) + GABA, the expression of SSADH in root, was also enhanced significantly as compared to the As(III) only except for As(III) + GABA(H) at short term. The results suggested that GABA with As(III) modulated the gene expressions of GABA shunt pathway to ameliorate the As(III) toxicity.

## Discussion

The elevated level of GABA regulates the antioxidant system and replenishes the intermediate of TCA cycle i.e. succinate during oxidative stress, through GABA shunt pathway^5, 8^. However, role of GABA against metalloid induced oxidative stress, particularly against As toxicity is still unclear in plants. The present study demonstrates the ameliorative effect of exogenous application of GABA against As stress other than GABA shunt pathway involving in plant tolerance mechanism.

Stress responsive signaling molecules are considered to play a crucial role in triggering stress-related genes expression in plants. Signaling molecule i.e. GABA and genes related to its metabolism enhances in the wide range of organisms like plants, mammals, algae and fungi etc. with diverse stress condition^30^. In present study, we observed that the expressions of Lsi-1 and Lsi-2 were enhanced with the As(III) treatments. However, GABA mainly reduced the expression of Lsi-1 than Lsi-2 against As(III) treatments, which decreased the accumulation of As in root and shoot. This might be due to the reduced gene expression Lsi-1 and Lsi-2 transporters in response to GABA signaling. The Lsi-1 is an influx transporter, facilitate the accumulation of As(III) from solution to root cells. This belongs to the Nod 26-like major intrinsic protein 2 (NIP2) subfamily of aquaporins. The Lsi-1 is mainly expressed in the root regions, where alteration of Lsi-1 transporter gene expression affect the accumulation of As(III) and silicon (Si)^26^. The Lsi-1 is more intensively expressed in the mature regions of the root. Conversely, the Lsi-2 is an efflux transporter, regulates the transportation of As(III) and Si from the root cells to the apoplast. The alteration in gene expression of Lsi-2 transporter can affect the translocation of As(III) and Si to the shoot/grain of rice plants^31^. In our study, the reduction in As accumulation corroborates with the earlier studies in which alteration of As accumulation in root and shoot was modulated with the Lsi-1 and Lsi-2 expression in rice^31^. Recently, the GABA application has been reported with the negative relationship of aluminium-activated malate transporter expression in *Triticum aestivum* ^2^ which coincide with suppression of Lsi-1 and Lsi-2 transporters gene expression with GABA application in our study. Plant contains several specific and non-specific transporters in the root for mineral transportation to the shoot. Further, the modulation in gene expression of these transporters can alter the transportation of elements. Through another study, it was shown that the higher expression of IRT1 transporter increased the accumulation of Cd and Fe in *Oryza sativa* ^32^, which strongly support our observations.

Biotic and abiotic stimuli, regulates the expression of GABA shunt pathway-associated genes and its concentration in plants results into enhanced expression of GABA shunt genes. In our study, the expressions of GABA shunt genes i.e. GAD 2, GAD 3, SSADH and SDH were found to be enhanced mainly in the roots with As(III) treatment. The enhanced expression of GABA shunt genes indicate that endogenous level of GABA provides higher tolerance in root than in shoots against As(III) toxicity. The enhanced expression of GAD genes against As(III) stress, in our study suggests a protective role of GABA shunt in rice plant through elevated level of GABA accumulation. In the GABA shunt, GAD is the important and first enzyme of the pathway and it modulates the endogenous level of GABA^5^. In response to As(III) toxicity, supplementation of GABA significantly enhanced the expression of GABA T2 and SSADH in shoots than in roots, which reveals that the exogenous GABA primarily provides shoot mediated tolerance in plants against As(III) toxicity. GABA-T and SSADH are two GABA catabolic enzymes, which involves in growth regulation and synthesis of succinate in TCA cycle through GABA shunt pathway. In order to adapt in adverse environmental conditions, plant up-regulates these enzymes largely for stress management^1^.

GABA is a plant growth promoting agent, plays crucial role in physiological characteristics when applied to the plants^2^. Our study reveals that the accumulation of GABA in both the exposure periods (Long term and short term) induces growth of rice seedlings. The growth was prominently increased with the long term than short term exposure. Increased in growth parameters (root, shoot and fresh weight) clearly reflects the growth promoting responses of GABA in the plants^33, 34^. The growth responses of GABA mainly depend upon its concentration in the plant tissues. In our study, long term exposure of GABA in plants exhibited more GABA accumulation than short term, which signifies the dose dependent responses of GABA in plants^29, 35, 36^. On the contrary, the treatment of 2 µg ml^−1^ As(III) reduced the growth parameters especially, shoot length and fresh weight, whereas, increased the root length of rice seedlings which was normalized with the GABA application. The exposure of As induces toxicity and inhibits the growth of plants^25^. However, it is reported that the exposure of As(III) and Cd upregulates the expression of the auxin-resistant gene AXR1 in *Arabidopsis* which facilitate the crosstalk between auxin and ethylene, involved in metal-induced root elongation^37^.

It is well established that, As(III) toxicity is associated with increased production of ROS followed by hampering the growth of plants due to its toxicity^25^. Treatments comprising of GABA supplemented with As(III), induced the growth which could be due to suppression of ROS generation, providing tolerance against metal stress^1^. The alteration of measured physiological parameters i.e. WUE, *A*, gs, PhiPS2, qp, NPQ, ETR and Trmmol, against As(III) stress, also recovered by supplementation with GABA. Congruent to our study, the alteration in net photosynthetic rate, stomatal conductance and internal CO_2_ concentration were also observed in cucumber plants against different metal stress (Cu, Cd and Pb)^38^. The physiological parameters regulate the different metabolic pathways of the plants. The physiological parameter WUE is the ratio of used water during plant metabolism to water lost through transpiration of plants and its alteration affects other interconnected mechanism. The alteration of WUE directly influences *A*, gs, ETR and Trmmol, which regulates the major mechanism such as, CO_2_ assimilation rate of photosynthesis, gaseous exchange, energy production and regulation of temperature of the plants. Our study reveals that the supplementation of GABA recovered all the measured physiological parameters against As(III) except NPQ. The marginal reduction in NPQ with GABA supplementation to As(III) treated seedlings, supports amelioration against stress. Overall, the study reveals that the growth and physiological parameters with GABA accumulation effective in reducing As(III) toxicity.

GABA is an important metabolite that limits the accumulation of ROS against oxidative stress and enhances antioxidant activities^1^. In present study, accumulation of GABA increased the activities of antioxidant enzymes (CAT, POD, GPX, SOD) against As(III) stress, which coincided with reduced levels of ROS and GSH/GSSG ratio in plant tissues. The long term treatments of GABA highly enhanced the antioxidant activities than short term. Along with GABA shunt pathway, GABA can also enhance antioxidant enzymes activities associated with oxidative injury to provide tolerance in plants^39^. On the contrary of present study, GABA was not found in the significant activation of SOD, CAT, APX, and GR activities against heat stress^40^. The GSH/GSSG ratio provides overall cellular redox status. The elevated level of GSH/GSSG ratio in plant tissues is the indication of increased oxidative stress^41^. The increased activities of antioxidant enzymes were also reported with GABA treatment against Al stress in barley seedlings^8^. In our study, rice plants supplemented with GABA to the As(III, showed the reduced level of TBARS and H_2_O_2_, which signifies the protective nature of GABA against As(III) stress. In present study, the TBARS content was decreased possibly be due to the GABA mediated reduction of As accumulation. Similarly, Li *et al*.^40^ reported GABA mediated reduction in TBARS content towards heat stress in *Agrostis stolonifera*. Arsenic is toxic metalloids, the elevated accumulation As enhances lipid peroxidation and ROS generation in plants, which disrupts physiological and biochemical process of the plants^25^. Thus, modulation of ROS and inhibition of lipid peroxidation through GABA, illustrates the augmentation of plant tolerance against As stress.

## Conclusion

The study provided evidence of GABA mediated suppression of Lsi-1 and Lsi-2 transporters genes against As toxicity, which is first to report in our knowledge. The elevated level of GABA accumulation proportionally decreased the accumulation of As and expressions of transporters (Lsi-1 and Lsi-2). In both the exposure periods, long term accumulation of GABA significantly reduced the expression of transporters and accumulation of As than short term, which sturdily strengthened plants against As toxicity. The GABA modulated antioxidant enzymes i.e., CAT, SOD, GPX and POD, participate in plants tolerance against As(III) induced stress. Overall, study suggested that GABA mediates stress tolerance via regulating the gene expression of transporters other than GABA shunt pathway genes towards As toxicity, which may propose a new understanding of GABA assisted plant responses.

## Methods

### Growth conditions and experimental design

The seeds of *Oryza sativa* L. cv. Pant-10 collected from Govind Ballabh Pant Agricultural University, Pantnagar, Uttrakhand, India were surface sterilized using 10% H_2_O_2_ for 5 min. Seeds were washed with double distilled water for removing H_2_O_2_ and spread on moist pre-sterilized blotting sheets in the tray. The trays containing seeds were kept in germinator maintained at 30 °C and 70% relative humidity. After 7 days (7 d), tray was placed in culture room maintaining light intensity at 210 µmol cm^−2^ sec^−1^ (16/8 h; day/night), produced from an array of cold fluorescent light at 24–26 °C temperature. The seedlings were allowed to grow for 15 d. After 15d of growth, 25 uniform size (10 cm) seedlings were placed in 150 ml glass beakers containing 100 ml of 100% Hewitt nutrient medium. The beakers were covered with black paper to avoid the exposure of light into the root and kept for 7 d to acclimatize. The treatments were made for As(III) (2 µg ml^−1^) and GABA (50 and 100 µg ml^−1^) using NaAsO_2_ and pure GABA (Sigma-Aldrich, USA), respectively. To examine time dependent responses of GABA, treatments were applied in two different exposure periods i.e., since germination (Long term) and for 7 d after acclimatization periods (Short term). The treatments of As(III) and As(III) along with GABA were provided to the seedlings for the 7 d after acclimatization.

For convenience, treatments and application have been abbreviated as, rice seedlings receiving 2 µg ml^−1^ of As(III) as As(III). The two concentration of GABA, 50 and 100 µg ml^−1^ [lower (L) and higher (H)] with different exposure periods i.e long term and short term abbreviated as GABA(L) (Long term), GABA(H) (Long term), GABA(L) (Short term) and GABA(H) (Short term), respectively. Similarly, 2 µg ml^−1^ of As(III) with different concentration and exposure period of GABA abbreviated as, As + GABA(L) (Long term), As + GABA(H) (Long term), As + GABA(L) (Short term) and As + GABA(H) (Short term), respectively. All the treatments were carried out in 4 replicates and harvested for analysis after 7 d of treatments.

### Extraction and derivatization of GABA for gas chromatographic analysis

The extraction of GABA content from plant samples was followed by the method of Weckwerth *et al*.^42^. To extract GABA, 200 mg of plant tissues were ground using liquid nitrogen until the fine powder with the help of chilled mortar and pestle. The ground tissues were dissolved in 1.5 ml of cold solvent containing methanol: chloroform: water in 5:2:1 ratio (stored at −20 °C). The homogenized plant tissues were kept on ice for 30 min with alternate shaking using orbital shaker. The homogenate was centrifuged at 12,000 rpm for 10 min and the supernatant was collected carefully. The collected samples were re-dissolved in deionized water and chloroform. The solution was vortex-mixed and centrifuged at 12, 000 rpm for 2 min for phase separation. The upper phase was collected carefully and dried in vaccum oven.

The vaccum dried samples were used for the derivatization process. The GABA was derivatized and measured by the method of Sobolevsky *et al*.^43^ using N-methyl-N-(tert-butyldimethylsilyl) trifluoroacetamide (MTBSTFA) silylating agent. 100 µl of acetonitrile and 100 µl of MTBSTFA were added in dried samples. The samples were heated at 70 °C for 30 min and 1 µl of derivatized sample was subjected to the gas chromatography. Samples were analyzed by the gas chromatography (Agilent GC model 7890 A) with FID using capillary BP-5 column (5% phenyl methyl polysiloxane column, 30 m × 0.32 mm × 0.25 µm). The injector and detector temperatures were maintained at 280 °C. The initial oven temperature was kept 70 °C for 2 min and increased to the final temperature of 300 °C with 5 °C/min.

### Gene expression analysis

In order to analyze the expression of genes, total RNA of shoot and root were extracted from 100 mg fresh weight tissues, using Plant RNeasy Kit (Qiagen), followed by treatment of RNase-free DNase I (Sigma-Aldrich, USA). Total RNA was converted into cDNA using Readyscript cDNA synthesis kit (Sigma-Aldrich, USA) according to manufacturer’s protocol. The primers were designed using primer3plus program: http//primer3plus.com. qRT-PCR was performed using 2 µl of cDNA corresponding to the set of selected genes and the reaction mixture containing 2 × PCR Mastermix (Thermo scientific, USA). The m-RNA levels were quantified using a Statagene Mx 3000 P qRT-PCR system (Agilent Technologies) with specific fluorescent dye SYBR green master mix (Thermo Fisher Scientific) using specific primers. Three technical replicates of each treatment were analyzed for qRT-PCR analysis. The primers of rice actin gene were used as an internal control to ensure that equal amounts of cDNA used in all the reactions. The PCR reaction was carried out using the following cycle conditions: an initial denaturation at 94 °C for 2 min, PCR cycles provided at 94 °C for 30 s, 55 °C for 30 s, and 72 °C for 30 s, followed by a final 5 min extension at 72 °C. After obtaining the Ct value for each reaction, the relative expression was calculated by ΔΔ^Ct^ method (Livak and Schmittgen^44^). The list of selected genes and oligonucleotide primers (Sigma-Aldrich, USA) used for each gene are listed in the additional file (Supplementary Table 1).

### Biochemical analysis

TBARS content was estimated spectrophotometrically at 532 and 600 nm following Hodges *et al*.^45^, using 6.65% thiobarbutric acid, 0.01% BHT mixed in 20% TCA and heated with sample at 95 °C for 45 min and H_2_O_2_ content by Velikova *et al*.^46^. The calculation was performed using ε = 0.155 M g^−1^ fw and deducting the turbidity recorded at 600 from 532. The ascorbic acid (µM g^−1^ fw) content was estimated spectrophotometrically at 525 nm following Kampfenkel *et al*.^47^ comparing it with the standard curve of L-ascorbic acid (Sigma-Aldrich, USA). Reduced and total glutathione (GSH and total GSH) were estimated by Anderson^48^ methods by estimating the stoichiometric formation of 5-thio-2-nitrobenzioc acid (TNB) at λ = 412 nm and comparing with a standard curve prepared with GSH (Sigma-Aldrich, USA).

For the analysis of enzyme activities, fresh leaves samples (300 mg each) were ground using liquid N_2_ in a chilled mortar and pestle and extracted with 3 ml chilled 100 mM potassium phosphate buffer (pH 7.5) containing EDTA (1 mM) and 1% (w/v) polyvinylpyrrolidone. The homogenate was centrifuged at 12,000 rpm for 15 min. Superoxide dismutase (SOD) (EC 1.15.1.1) activity was measured spectrophotometrically at 560 nm following Beauchamp & Fridovich^49^ and presented as U mg^−1^ protein, where 1 U of SOD activity is the amount of protein required to inhibit 50% of initial reduction of nitro-blue tetrazolium (NBT) under light. Ascorbate peroxidase (APX) (EC 1.11.1.11) activity was measured following Nakano & Asada^50^ using ε = 2.8 mM^−1^ cm^−1^ and enzyme activity was expressed as µM of ascorbate oxidized min^−1^ mg^−1^ protein. Catalase (CAT) (EC 1.11.1.6) activity was measured by the method of Chandlee & Scandalios *et al*.^51^ and expressed as mM min^−1^ mg^−1^ protein. Guaiacol peroxidase (POD) (EC 1.11.1.7) activity was measured at 470 nm following Kato & Shimizu^52^ using ε = 26.6 mM^−1^ cm^−1^ and expressed as µM of guaiacol oxidized min^−1^ mg^−1^ protein. Glutathione reductase (GR) (EC 1.6.4.2) activity was assayed following Smith *et al*.^53^ and represented as U mg^−1^ protein, where 1U is the conversion of 1 mM of oxidized glutathione (GSSG) min^−1^ into reduced glutathione (GSH). Ascorbate oxidase (AO) (EC 1.10.3.3) activity was assayed following Oberbacher & Vines^54^ and represented as U mg^−1^ protein, where 1U is the oxidation of 1 µM of the ascorbic acid min^−1^. Monodehydroascorbate reductase (MDHAR; EC 1.6.5.4) activity was assayed following Vanaker *et al*.^55^ by monitoring the formation of monodehydroascorbate at λ = 340 nm (1 mM of ascorbate oxidized per min) and represented as mM mg^−1^ protein. Dehydroascorbate reductase (DHAR; EC 1.8.5.1) activity was measured by Doulis *et al*.^56^ at λ = 265 nm using ε = 7.0 mM^−1^ cm^−1^ and represented as µM mg^−1^ protein. Glutathione peroxidase (GPX) activity was measured by Takeda *et al*.^57^ a total volume of 1 ml reaction mixture containing 100 mM Tris-HCl (pH-7.5), 1 mM GSH, 0.4 mM NADPH, 0.2 mM H_2_O_2_ and 1 unit of GR. The depletion of NADPH is monitored at 340 nm, which reduces H_2_O_2_ to H_2_O. Glutathione S-transferase (GST; EC 2.5.1.13) activity was measured by Habig *et al*.^58^ using ε = 9.6 mM^−1^ cm^−1^ of the product and expressed as µM of CDNB (1-Chloro-2, 4-dinitrobenzene) conjugated mg^−1^ protein.

### Physiological and growth parameters

The effects of different treatments on the physiological parameters of the cultivar Pant-10 plants were evaluated by an open flow gas exchange system (Li-Cor 6400XT; Li-Cor, Inc., Lincoln, NE, USA) for 7 d old plantlets from treatments. The air flow rate, relative humidity, T_leaf_, VPD, and CO_2_ conc. were kept constant at 55%, 28 °C, 1.5 KPa and 400 µmol m^−2^ sec^−1^ respectively. The plantlets of the cultivar were subjected to saturating PPFD of 1200 µM m^−2^ sec^−1^ (provided by artificial LED light source Li-Cor 6400-02B). Prior to beginning of each measurement, the leaves were dark adapted by using dark adapting clips (Li-Cor 9964-091) for 20 minutes. The fresh-weight (mg), root and shoot lengths (cm) were measured after 7 d of treatments by an electrical balance (Mettler-Toledo) and metric scale.

### Elemental analysis

The content of total As was determined in acid digested sample by AAS (GBC Avanta ∑) hydrite generator (MDS 2000) using NaH_2_BO_4_ (1.5 mM) + NaOH (1.5 mM) and HCl (3 mM). The accuracy of the instrument was ensured by checking the values of standard reference material (CRM029-050) (Supplementary Table 2).

### Statistical analysis

All the values are average of four replicates. The data were subjected to Duncan’s Multiple Range Test (DMRT) for the analysis of significant difference between the means (p < 0.05). All the values are represented as percentage increase or percentage decrease with respect to the respective values in control seedlings, or otherwise mentioned.

## Electronic supplementary material

Supplementary Table 1

Supplementary figure 1

Supplementary figure 2

Supplementary table 2
