# Supplementary material for: GABA accretion reduces Lsi-1 and Lsi-2 gene expressions and modulates physiological responses in Oryza sativa to provide tolerance towards arsenic
Source: Sci Rep. 2017 Aug 18;7:8786. doi: 10.1038/s41598-017-09428-2 (PMC5562799; doi:10.1038/s41598-017-09428-2)
Supplement: Supplementary file 1 — Supplementary Table 1 [file 41598_2017_9428_MOESM1_ESM.doc]

**GABA accretion reduces Lsi-1 and Lsi-2 gene expressions and modulates physiological responses in *Oryza sativa* to provide tolerance towards arsenic**

Navin Kumar^1,2^, Arvind Kumar Dubey^1^, Atul Kumar Upadhyay^1^, Ambedkar Gautam^1#^, Ruma Ranjan^1#^, Saripella Srikishna^2^, Nayan Sahu^1^, Soumit Kumar Behera^1^, Shekhar Mallick^1^*

^1^ CSIR-National Botanical Research Institute, Lucknow, India

^2^Department of Biochemistry, Faculty of Science, Banaras Hindu University, Varanasi, India

^#^ These authors contributed equally to this work

* Author for correspondence:

Dr. Shekhar Mallick

Email: [shekharm@nbri.res.in](mailto:shekharm@nbri.res.in), Phone: 0522-2297847

| **Locus Id.** | **Primer sequences** | **Gene Name** |
| --- | --- | --- |
| LOC_Os03g13300 | F_GTGGAGCTCAAGGAGGTGAA  R_CGAGGAGGTCGTTGAGTAGC | GAD 1 |
| LOC_Os03g51080 | F_TGCACACCTCTTCAATGCTC  R_CTCCCAGCAAACTTGGACAT | GAD 2 |
| LOC_Os04g37460 | F_TGGTGAAGAGCATCAACGTC  R_GGAGAAGTTGAGCGTGAAGG | GAD 3 |
| LOC_Os04g52450 | F_TTTGGACCCATTGGACAT  R_CGAGGGATTATTACTCTGTACATCC | GABA T1 |
| LOC_Os04g52440 | F_CCGCTGTCGTTGTTGTAGTGAT  R_CGCATCTAGAACAAGCTTCAGG | GABA T2 |
| LOC_Os02g0173900 | F_TGCTGAAGAAGCAAAGCGTA  R_CTCTGATGGCTTGACAACGA | SSADH |
| LOC_Os02g40830 | F_ATCAGAGGGACAGGTTGTGG  R_ACAGGCACCTTCAGGTCAAC | S.CoA ligase |
| LOC_Os02g31040.1 | F_TTGGCTGAGGGATAGGATTG  R_ATGCCAGGGATCAGTGTCTC | α-KDH |
| LOC_Os09g20440 | F_AAGGAGCCCATTCCTGTCTT  R_GAATTTGCACCCAGACGATT | SDH |
| LOC_Os02g0745100 | F_CGGTGGATGTGATCGGAACCA  R_CGTCGAACTTGTTGCTCGCCA | Lsi-1 |
| LOC_Os03g0107300 | F_CAACACGATGATGTCGCTGG  R_CACGTACGCGAAGCTCTTGA | Lsi-2 |
| Rice Actin | F_GAGTATGATGAGTCGGGTCCAG  R_ACACCAACAATCCCAAACAGAG | Actin |

**Supplemental Table 1.** Primer sequence of GABA shunt, TCA cycle and As(III) specific transporters for gene expressions analysis in *Oryza sativa* L.
