# Supplementary material for: GABA accretion reduces Lsi-1 and Lsi-2 gene expressions and modulates physiological responses in Oryza sativa to provide tolerance towards arsenic
Source: Sci Rep. 2017 Aug 18;7:8786. doi: 10.1038/s41598-017-09428-2 (PMC5562799; doi:10.1038/s41598-017-09428-2)
Supplement: Supplementary file 2 — Supplementary figure 1 [file 41598_2017_9428_MOESM2_ESM.doc]

**GABA accretion reduces Lsi-1 and Lsi-2 gene expressions and modulates physiological responses in *Oryza sativa* to provide tolerance towards arsenic**

Navin Kumar^1,2^, Arvind Kumar Dubey^1^, Atul Kumar Upadhyay^1^, Ambedkar Gautam^1#^, Ruma Ranjan^1#^, Saripella Srikishna^2^, Nayan Sahu^1^, Soumit Kumar Behera^1^, Shekhar Mallick^1^*

^1^ CSIR-National Botanical Research Institute, Lucknow, India

^2^Department of Biochemistry, Faculty of Science, Banaras Hindu University, Varanasi, India

^#^ These authors contributed equally to this work

* Author for correspondence:

Dr. Shekhar Mallick

Email: [shekharm@nbri.res.in](mailto:shekharm@nbri.res.in), Phone: 0522-2297847

**
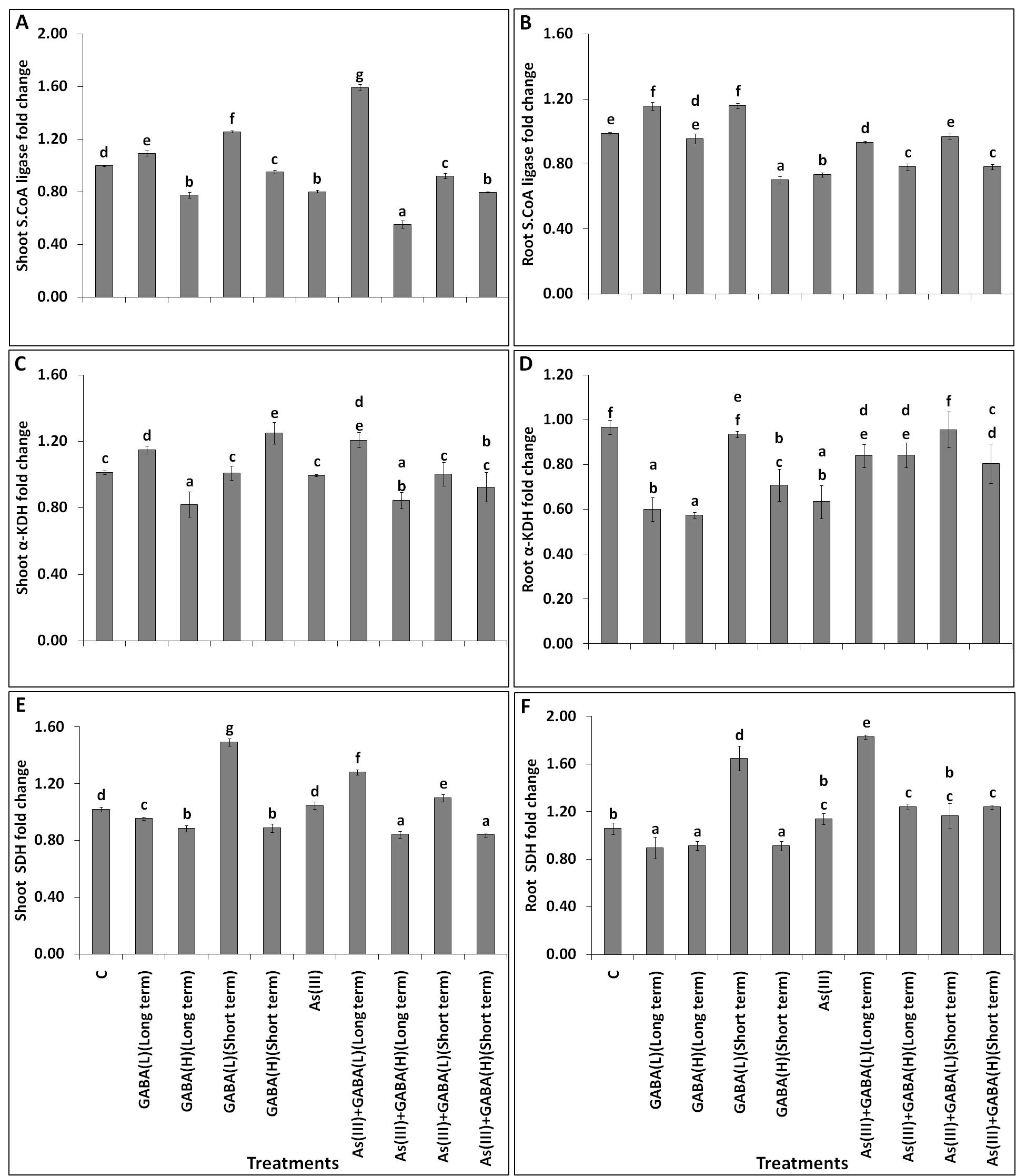
**

**Supplementary Fig. 1.** Oxidative stress sensitive different genes expressions of TCA cycle enzymes [S.CoA ligase: Succinyl CoA ligase (A, B); α-KDH: α-Ketoglutarate dehydrogenase (C, D); SDH: Succinate dehydrogenase (E, F)] in *Oryza sativa* L. under different combinations of As(III) and GABA. All the values are presented as mean ± SD (n=4).
