# Supplementary material for: GABA accretion reduces Lsi-1 and Lsi-2 gene expressions and modulates physiological responses in Oryza sativa to provide tolerance towards arsenic
Source: Sci Rep. 2017 Aug 18;7:8786. doi: 10.1038/s41598-017-09428-2 (PMC5562799; doi:10.1038/s41598-017-09428-2)
Supplement: Supplementary file 3 — Supplementary figure 2 [file 41598_2017_9428_MOESM3_ESM.doc]

**GABA accretion reduces Lsi-1 and Lsi-2 gene expressions and modulates physiological responses in *Oryza sativa* to provide tolerance towards arsenic**

Navin Kumar^1,2^, Arvind Kumar Dubey^1^, Atul Kumar Upadhyay^1^, Ambedkar Gautam^1#^, Ruma Ranjan^1#^, Saripella Srikishna^2^, Nayan Sahu^1^, Soumit Kumar Behera^1^, Shekhar Mallick^1^*

^1^ CSIR-National Botanical Research Institute, Lucknow, India

^2^Department of Biochemistry, Faculty of Science, Banaras Hindu University, Varanasi, India

^#^ These authors contributed equally to this work

* Author for correspondence:

Dr. Shekhar Mallick

Email: [shekharm@nbri.res.in](mailto:shekharm@nbri.res.in), Phone: 0522-2297847

**Supplementary Fig. 2.** Effect of GABA accumulation on (A) Stomatal conductance (gs), (B) Fraction of absorbed photons used in photochemistry (PhiPS2), (C) Photochemical quenching (qP) and (D) Electron transport rate (ETR) in the leaves of the *Oryza sativa* L. with As(III) treatments. Values marked with same alphabets are not significantly different (DMRT, p<0.05). All the values are means of four replicates ±SD.
