# Supplementary material for: GABA accretion reduces Lsi-1 and Lsi-2 gene expressions and modulates physiological responses in Oryza sativa to provide tolerance towards arsenic
Source: Sci Rep. 2017 Aug 18;7:8786. doi: 10.1038/s41598-017-09428-2 (PMC5562799; doi:10.1038/s41598-017-09428-2)
Supplement: Supplementary file 4 — Supplementary table 2 [file 41598_2017_9428_MOESM4_ESM.doc]

**GABA accretion reduces Lsi-1 and Lsi-2 gene expressions and modulates physiological responses in *Oryza sativa* to provide tolerance towards arsenic**

Navin Kumar^1,2^, Arvind Kumar Dubey^1^, Atul Kumar Upadhyay^1^, Ambedkar Gautam^1#^, Ruma Ranjan^1#^, Saripella Srikishna^2^, Nayan Sahu^1^, Soumit Kumar Behera^1^, Shekhar Mallick^1^*

^1^ CSIR-National Botanical Research Institute, Lucknow, India

^2^Department of Biochemistry, Faculty of Science, Banaras Hindu University, Varanasi, India

^#^ These authors contributed equally to this work

* Author for correspondence:

Dr. Shekhar Mallick

Email: [shekharm@nbri.res.in](mailto:shekharm@nbri.res.in), Phone: 0522-2297847

| **Supplementary Table 2**. Recovery results of the Standard Reference Material (CRM 028-050) procured from Resource Technology Corporation (Lot No. IH 028) with As level 3.83 mg Kg^-1^. | | | | | | |
| --- | --- | --- | --- | --- | --- | --- |
| Sl No. | Wt. (g) | Final Vol. (ml) | Dilution Factor | µg Kg^-1^ | mg Kg^-1^ | % error |
| 1 | 0.302 | 10 | 6 | 16.13 | 3.20 | 15.66 |
| 2 | 0.3 | 10 | 6 | 15.31 | 3.36 | 11.46 |
| 3 | 0.295 | 10 | 6 | 14.34 | 3.84 | -1.21 |
| 4 | 0.321 | 10 | 6 | 16.32 | 3.61 | 4.99 |
| 5 | 0.331 | 10 | 6 | 18.06 | 3.52 | 7.17 |
| 6 | 0.326 | 10 | 6 | 14.39 | 2.97 | 21.70 |
| 7 | 0.384 | 10 | 6 | 16.67 | 2.93 | 22.86 |
| 8 | 0.291 | 10 | 6 | 17.32 | 3.53 | 7.17 |
| 9 | 0.289 | 10 | 6 | 15.36 | 3.45 | 9.17 |
| 10 | 0.334 | 10 | 6 | 18.36 | 3.95 | -3.98 |
